# Genes, microbiome, diet and inflammatory bowel disease

**DOI:** 10.1186/1824-7288-41-S2-A4

**Published:** 2015-09-30

**Authors:** Robert N Baldassano

**Affiliations:** 1 Children's Hospital of Philadelphia, University of Pennsylvania, Philadelphia, PA 19104, USA

## Background

The incidence of inflammatory bowel diseases (IBD), including Crohn's disease (CD) and ulcerative colitis (UC) is increasing worldwide [1]. These diseases result in chronic, relapsing inflammation of the gastrointestinal tract. The pathogenesis of IBD is currently thought to involve an inappropriate and persistent inflammatory response to commensal gut microbes in genetically susceptible individuals. Advances in DNA sequencing technology have led to the association of > 163 genetic polymorphisms with risk for IBD. . However, in total, these loci only account for about 13% of CD and 7% of UC disease variance. Therefore, it appears that environmental factors make the largest contributions to IBD risk. Among the environmental factors associated with IBD, diet and the intestinal microbiota are the most likely to be modifiable making them targets for prevention and treatment of IBD.

While nutritional therapy has been shown to be efficacious in the treatment of CD, the mechanism of action has not been well characterized. Some hypotheses involve reduction in luminal antigens and food exclusion, a direct anti-inflammatory effect of the formula, improved nutrition, and changes in the gut microbiota [2-5]. The discovery that formula composition does not impact outcome somewhat opposes the hypothesis that enteral nutritional therapy is delivering a substance that is beneficial to the gastrointestinal tract. A recent study completed at our institution showed exclusive enteral nutrition was similar to anti-TNF therapy for induction of remission but partial enteral nutrition was inferior to these therapies. Our data suggest that EEN is likely effective based on exclusion of a “harmful” factor rather than through more effective delivery of a specific nutrient.

Modulation of the gut microbiota composition is a proposed mechanism of action of enteral nutritional therapy, although the current data are sparse [6]. The available literature on this subject suggests that there is a profound change in the fecal microbiota following EEN therapy [3,6].

## Conclusions

In summary, it is clear that enteral nutritional therapy is a safe and effective approach to the treatment of Crohn's disease. Induction of remission and healing of the intestinal mucosa can be accomplished with enteral nutritional therapy. Enteral nutritional therapy may also be effective in maintaining remission and preventing post-operative recurrence of disease following resection. An enhanced understanding of the mechanism of action may allow for the development of less restrictive protocols which achieve the same effect. Additionally, mechanistic studies may help to identify patient populations who may be more likely to respond.

## References

[B1] Molodecky NA Soon IS Rabi DM Ghali WA Ferris M Chernoff G Increasing incidence and prevalence of the inflammatory bowel diseases with time, based on systematic review Gastroenterology 2012 142 46 54 e42 quiz e30 10.1053/j.gastro.2011.10.001 22001864

[B2] Gassull MA Review article: the role of nutrition in the treatment of inflammatory bowel disease Aliment Pharmacol Ther 2004 20 Suppl 4 79 83 15352899 10.1111/j.1365-2036.2004.02050.x

[B3] Lionetti P Callegari ML Ferrari S Cavicchi MC Pozzi E de Martino M Enteral nutrition and microflora in pediatric Crohn's disease JPEN J Parenter Enteral Nutr 2005 29 4 Suppl S173 5 discussion S175-8, S184-8 10.1177/01486071050290S4S173 15980280

[B4] van den Bogaerde J Kamm MA Knight SC Immune sensitization to food, yeast and bacteria in Crohn's disease Aliment Pharmacol Ther 2001 15 1647 53 10.1046/j.1365-2036.2001.01032.x 11564006

[B5] Van Den Bogaerde J Cahill J Emmanuel AV Vaizey CJ Talbot IC Knight SC Gut mucosal response to food antigens in Crohn's disease Aliment Pharmacol Ther 2002 16 1903 15 10.1046/j.1365-2036.2002.01360.x 12390099

[B6] Leach ST Mitchell HM Eng WR Zhang L Day AS Sustained modulation of intestinal bacteria by exclusive enteral nutrition used to treat children with Crohn's disease Aliment Pharmacol Ther 2008 28 724 33 10.1111/j.1365-2036.2008.03796.x 19145728

